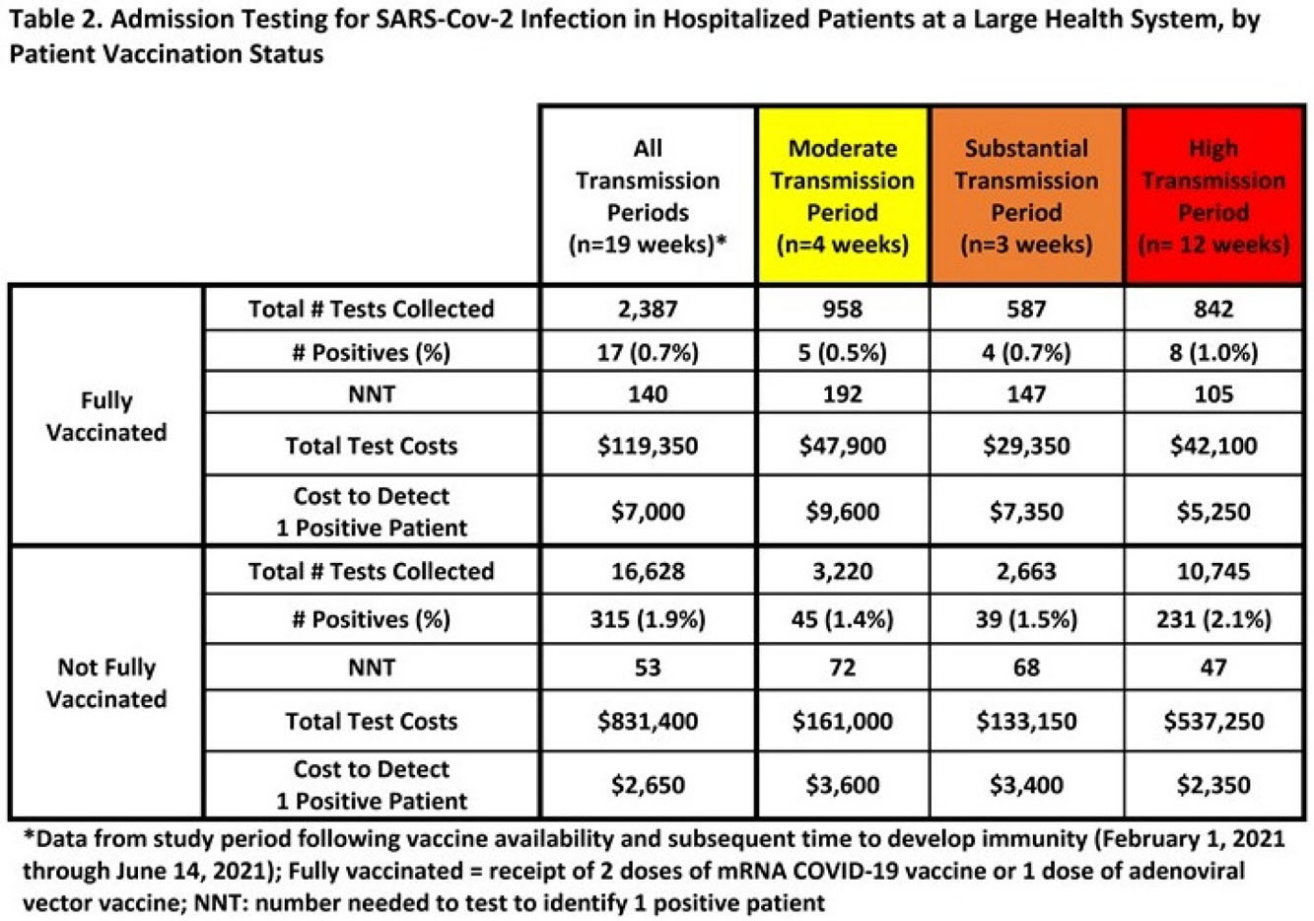# Analysis of Universal admission laboratory screening for SARS-CoV-2 asymptomatic infection across a large health system

**DOI:** 10.1017/ash.2022.72

**Published:** 2022-05-16

**Authors:** Jennifer Cihlar, Bryan Harris, Patty Wright, Romney Humphries, Kelly (Caroline), Brandi Cherry, Thomas Talbot

## Abstract

**Background:** Admission laboratory screening for asymptomatic COVID-19 has been utilized to mitigate healthcare-associated SARS-CoV-2 transmission. A better understanding of the impact of such testing across a variety of patient populations is needed. **Methods:** Beginning April 2020, every patient admitted within an academic healthcare system underwent SARS-CoV-2 PCR testing upon admission. Between April 20, 2020 through June 14, 2021, results were analyzed in asymptomatic patients across 4 inpatient facilities: a tertiary-care adult hospital, a free-standing pediatric hospital, a community-based hospital, and a behavioral health hospital. Positivity rates and the number needed to test (NNT) to identify 1 asymptomatic infected patient were calculated overall, by hospital type, by patient vaccination status, and by CDC-defined levels of community transmission. Weekly community incidence rates of COVID-19 for the system’s metropolitan service area (8 central Tennessee counties) were obtained from Tennessee Department of Health records. Weekly COVID-19 incidence rates per 100,000 people were calculated using US Census Bureau data. Using a national survey of hospital epidemiologists, a clinically meaningful NNT was identified (ie, 1 positive patient per 100 patients tested). A crude admission testing cost (covering testing supplies, reagents, and lab personnel costs) was obtained from operational data ($50 per test) to assess testing utility. **Results:** In total, 51,187 tests were collected during the study period with a positivity rate of 1.8%. No periods of low transmission were observed (Table 1). During high transmission periods, the NNT met the clinically relevant threshold in all populations. In addition, the NNT approached or met the 1:100 threshold for most locations during periods of less transmission, suggesting continued benefit even as infection rates decline. In all transmission periods, the NNT for non–fully vaccinated patients met the clinically meaningful threshold, in contrast to testing of fully vaccinated patients (Table 2). **Discussion:** Implementing an asymptomatic patient admission testing program can provide clinically relevant value based on the NNT, even during lower periods of transmission and in different patient populations. Limiting admission testing to non–fully vaccinated patients during periods of lower transmission may be a strategy to address cost and resource concerns around this practice. Further investigations into the impact of booster vaccination and newer SARS-CoV-2 variants on admission testing programs are also necessary. Although the impact of such testing on healthcare-associated COVID-19 among patients and healthcare workers could not be clearly determined, these data provide important information as facilities weigh the costs and benefits of such testing.

**Funding:** None

**Disclosures:** None

**Table:**
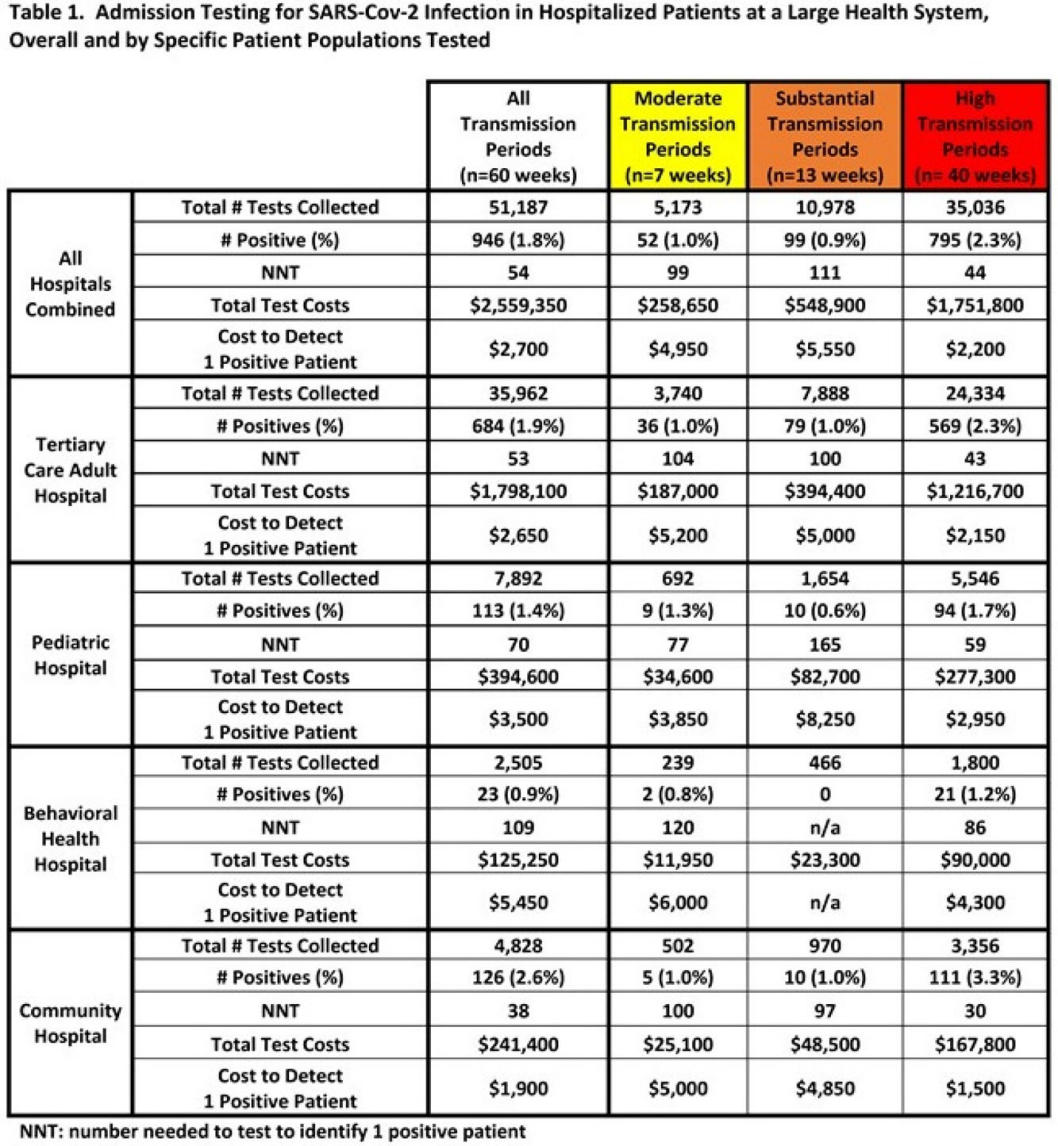

**Table:**